# Titers of Neutralizing Antibodies against SARS-CoV-2 Are Independent of Symptoms of Non-Severe COVID-19 in Young Adults

**DOI:** 10.3390/v13020284

**Published:** 2021-02-12

**Authors:** Hulda R. Jonsdottir, Michel Bielecki, Denise Siegrist, Thomas W. Buehrer, Roland Züst, Jeremy W. Deuel

**Affiliations:** 1 SPIEZ Laboratory, Austrasse, 3700 Spiez, Switzerland; Hulda-Run.Jonsdottir@babs.admin.ch (H.R.J.); Denise.Siegrist@babs.admin.ch (D.S.); 2 Swiss Armed Forces, Medical Services, Worblentalstrasse, 3063 Ittigen, Switzerland; eudosson@gmail.com (M.B.); Thomas.Buehrer@vtg.admin.ch (T.W.B.); 3 Epidemiology, Biostatistics and Prevention Institute, Travel Clinic, Hirschengraben, University of Zurich, 8006 Zürich, Switzerland; 4 Jeffrey Cheah Biomedical Centre, Department of Haematology and MRC—Wellcome Stem Cell Institute, Puddicombe Way, University of Cambridge, Cambridge CB2 AW, UK

**Keywords:** SNT, virus neutralization test, ELISA, young adults, SARS-CoV-2, COVID-19

## Abstract

Neutralizing antibodies are an important part of the humoral immune response to SARS-CoV-2. It is currently unclear to what extent such antibodies are produced after non-severe disease or asymptomatic infection. We studied a cluster of SARS-CoV-2 infections among a homogeneous population of 332 predominantly male Swiss soldiers and determined the neutralizing antibody response with a serum neutralization assay using a recombinant SARS-CoV-2-GFP. All patients with non-severe COVID-19 showed a swift humoral response within two weeks after the onset of symptoms, which remained stable for the duration of the study. One month after the outbreak, titers in COVID-19 convalescents did not differ from the titers of asymptomatically infected individuals. Furthermore, symptoms of COVID-19 did not correlate with neutralizing antibody titers. Therefore, we conclude that asymptomatic infection can induce the same humoral immunity as non-severe COVID-19 in young adults.

## 1. Introduction

To date, more than 100 million people have been infected by the novel zoonotic coronavirus (CoV), Severe Acute Respiratory Syndrome (SARS)-CoV-2, and over two million have succumbed to the disease. 

The disease caused by SARS-CoV-2, Coronavirus Disease 2019 (COVID-19), presents with a wide range of symptoms of varying severity. Although the virus can infect and cause disease in humans of all ages, disease severity seems to correlate with older age [1,2,3]. Frequent asymptomatic SARS-CoV-2 infections have been described, complicating both the treatment and containment of the virus [4,5,6]. The humoral immune response to viral infections is characterized by the generation of virus-specific antibodies (immunoglobulins, Ig) and the frequent occurrence of virus-neutralizing antibodies (nAb) [7]. However, many essential aspects of virus–host interactions during SARS-CoV-2 infection remain unclear, including the production and longevity of virus-specific antibodies in infected persons, and their implications on protection from re-infection. At present, there are conflicting data on the incidence of virus-neutralizing antibodies in asymptomatic and non-severe cases of COVID-19 [8,9]. Given the range of disease severity in humans, it is important to elucidate the similarities and differences between symptomatic and asymptomatic infections of SARS-CoV-2 and their implications for long-term protection, potential re-infections, herd immunity, and treatment options. In the current study, we utilized a fully infectious, recombinant clone of SARS-CoV-2 expressing green fluorescent protein (GFP) [10] to compare the neutralizing capacity of patients with non-severe COVID-19 to asymptomatic young adults in a large homogenous cohort. We show that titers of antibodies preventing cellular infection by SARS-CoV-2-GFP are similar between asymptomatically infected and symptomatic patients.

## 2. Materials and Methods

### 2.1. Cohort

**Figure 1:**
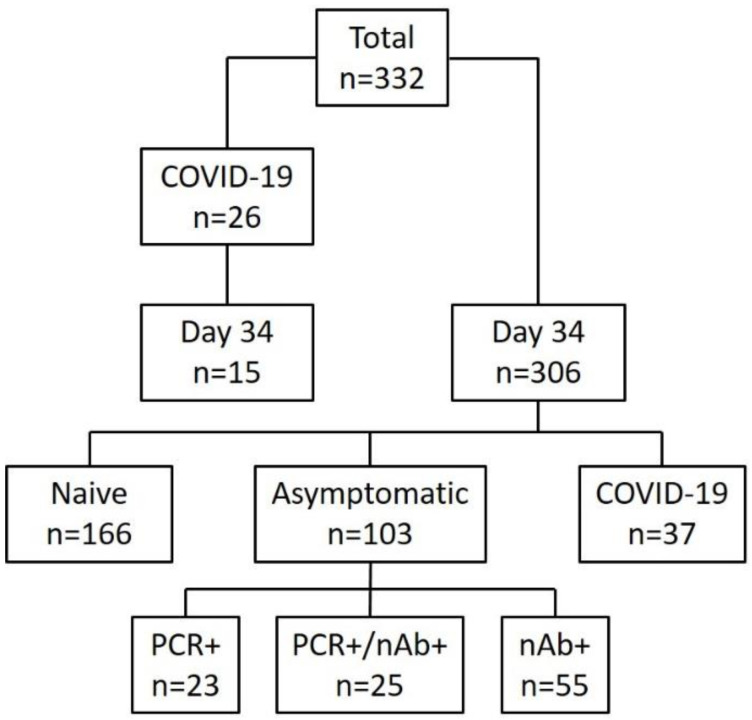
Schematic overview of the study. A total of 332 individuals participated: 306 in the cross-sectional part (one single sampling on 14 April 2020) and 26 patients suffering from non-severe COVID-19 in the longitudinal part with daily sampling. Out of 306 participants, 37 (12%) had previously been diagnosed with COVID-19, 103 (32%) had evidence of asymptomatic infection and 166 had no evidence of infection by SARS-CoV-2. Of the 103 asymptomatically infected, 23 were solely positive by PCR but had no neutralizing antibodies (nAbs), 55 (53%) had solely nAbs but were PCR negative and 25 (24%) had both, a positive PCR and nAb.

The entire cohort consisted of 584 young Swiss soldiers with a median age of 21; 332 individuals participated in this study. Twenty-six soldiers diagnosed with COVID-19 between 11 March and 7 April 2020, were included in the study’s longitudinal part. These patients were assessed daily for symptoms, vital parameters, and infectious status via questionnaire, physical examination and nasopharyngeal swabbing. We also obtained serum samples on the day of diagnosis, and 7 and 14 days after the onset of symptoms. On 14 April 2020, 34 days after the first confirmed case, we conducted cross-sectional testing of 321 soldiers; they were screened for SARS-CoV-2 with qRT-PCR and a serum sample obtained simultaneously. We also asked participants to complete an electronic questionnaire. Of these 321 subjects, 52 (16%) had previously been diagnosed with COVID-19 (median time since diagnosis: 23 days, range of 9–35 days), of which 15 were also included in the longitudinal study. All participants were followed up until 3 May 2020 (i.e., for an additional 19 days), and no new cases of COVID-19 were observed. A schematic overview of the cohort is presented in Figure 1. 

### 2.2. Clinical Observations

Peripheral oxygen saturation was measured by finger-clip oximetry using the index finger. Temperature was acquired tympanically with a ThermoScan Instrument (Braun, Sempach, Switzerland). Every soldier was required to immediately present at theclinic if feeling unfit for service or with symptoms of COVID-19. These were immediately isolated and tested for SARS-CoV-2 by a deep nasopharyngeal swab and RT-qPCR. Symptoms were assessed during cross-sectional testing with an online questionnaire and were, thus, self-reported.

### 2.3. Virus Propagation

Recombinant SARS-CoV-2-GFP [10] was propagated on Vero E6 cells cultured in minimal essential medium (MEM, Seraglob, Schaffhausen, Switzerland), supplemented with 2% heat inactivated fetal bovine serum (FBS, Biochrom AG, Berlin, Germany) and 100 U/mL penicillin/streptomycin (Seraglob, Schaffhausen, Switzerland) at 37 °C, 5% CO_2_ and >85% relative humidity (rH) for 72 h prior to harvest. Subsequently, the infectious titer was determined by endpoint dilution and calculation of the tissue culture infectious dose 50 (TCID_50_) under the same conditions. The recombinant SARS-CoV-2-GFP and Vero E6 cells were provided by Prof. Volker Thiel, University of Bern, Switzerland. All experiments involving infectious SARS-CoV-2 followed the approved standard operating procedures of the biosafety level 3 (BSL-3) facility at SPIEZ Laboratory.

### 2.4. Serological Assays

Immunoglobulins (Ig) M, G (against SARS-CoV-2 N, EDI Epitope Diagnostics Inc, San Diego, CA, USA) and A (against SARS-CoV-2 S1, Euroimmun Medizinische Labordiagnostika AG, Lübek, Germany) were analyzed by Enzyme-linked immunosorbent assay (ELISA) as previously described [4]. The presence of total antibodies against the SARS-CoV-2 nucleocapsid was determined by electrochemiluminescence immunoassay (ECLIA, (Elecsys^®^), Roche Diagnostics AG, Rotkreuz, Switzerland) using Cobas e411 according to the manufacturer’s instructions.

### 2.5. Serum Neutralization Test (SNT)

Participant serum samples were inactivated for 30 min at 56 °C and subsequently diluted 1:5 in MEM + 2% FBS (Seraglob, Schaffhausen, Switzerland). Further 5-fold dilutions were made in a 96-well plate (Techno Plastic Products (TPP), Trasadingen, Switzerland) in a total volume of 100 µL. One hundred TCID_50_ SARS-CoV-2-GFP were added in equal volume. Sera and virus were incubated for 1 h at 37 °C before transfer to confluent Vero E6 cells. Cells were then incubated at 37 °C, 5% CO_2_ and >85% rH for 72 h. After incubation, neutralization capacity was evaluated by crystal violet staining and reported as the geometric mean titer (GMT) of three replicates.

### 2.6. RT-qPCR

To verify a laboratory confirmed infection, RNA from nasal swabs was extracted on a MagNA Pure 96 instrument (Roche, Rotkreuz, Switzerland) and real-time qPCR was performed in triplicate for SARS-CoV-2 as previously described [4]. Five microliters of eluted RNA was analyzed with the TaqMan Fast Virus 1-step Master Mix (Applied Biosystems, Fisher Scientific AG, Reinach, Switzerland).

The SARS-CoV-2 Envelope (E) gene was detected by these oligonucleotides (TIB Molbio, Berlin, Germany):

fwd primer: 5′-ACAGGTACGTTAATAGTTAATAGCGT-3′, 

probe: 5′-FAM-ACACTAGCCATCCTTACTGCGCTTCG-BBQ-3′ 

rev primer: 5′-ATATTGCAGCAGTACGCACACA-3′ [11]

The nsp12 gene was detected by the following oligonucleotides (Thermo Fisher AG, Zug, Switzerland): 

fwd primer 5′-CCACGCCAGGTAGTGGAGTT-3′ 

probe: 5′-FAM-CTATATTAACCTTGACCAGGGC-MGB-3′ 

rev primer: 5′-AAGGCTTTGTTAAGTCAGTGTCAACA-3′ 

Cycling parameters: 5 min at 50 °C, 20 s at 95 °C followed by 45 cycles of 3 s at 95 °C and 30 s at 60 °C.

### 2.7. Graphical Representation and Statistical Analysis

Data were analyzed and visualized with the R statistical Software (R Core Team, Vienna, Austria: www.r-project.org, accessed on 22 January 2021) version 3.6.1.

## 3. Results

### 3.1. Neutralizing Antibody Titers Do Not Decline within One Month Post COVID-19 Diagnosis

**Figure 2:**
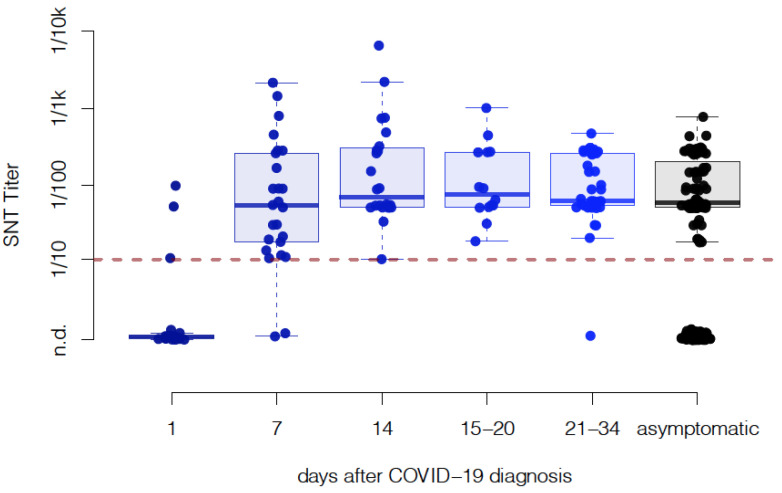
Serum neutralizing antibody titers of mildly symptomatic and asymptomatic individuals. Neutralizing titers of patients with non-severe COVID-19 and asymptomatically infected individuals were measured in quadruplicate and shown as the geometric mean titer (GMT). No statistically significant difference in titers was found between asymptomatic participants with a detectable titer and symptomatic patients at or after day 14 by the Wilcoxon rank sum test with continuity correction (*p* = 0.77). Healthy volunteers who either tested positive for viral RNA in nasal swabs or had detectable nAbs were defined as asymptomatic. Each dot represents one individual. n.d. = not detectable.

For the longitudinal study, a serum sample was drawn from 26 COVID-19 patients on the day of diagnosis (day 1) and days 7, and 14 after the onset of symptoms. Additionally, a cross-sectional sampling including 37 patients that had previously suffered from COVID-19, along with 15 patients previously included in the longitudinal study, was conducted and grouped into samples from days 14–20 (*n* = 14) and 21–34 (*n* = 38) post diagnosis (Figure 2). All patients had a predominantly non-severe course and were treated symptomatically. No patient died, was admitted to the intensive care unit, or needed mechanical ventilation. Seven days post diagnosis, 92% of patients had developed detectable neutralizing antibodies (nAbs) (median titer 1:50, min not detected, max 1:2137, *n* = 23) and after 14 days, 100% had developed nAbs (median titer 1:68, min 1:10, max 1:6250, *n* = 24). Between days 15 and 20, all patients had a detectable titer (median titer: 1:68, min 1:17, max 1:1000, *n* = 14) and between days 21 and 34, 97% of patients had detectable nAb (median titer 1:50, min not detected, max 1:430, *n* = 38). Thus, the sustained titers of neutralizing antibodies against SARS-CoV-2 were detected for a month after COVID-19 diagnosis.

### 3.2. Cumulative Neutralizing Capacity of Asymptomatic Cases Is Similar to Symptomatic Patients

We cross-sectionally sampled 321 soldiers, of which 269 (84%) had never been diagnosed with COVID-19 and did not develop symptoms of COVID-19 in the 19 days following sampling. Of these 269 individuals, 80 (29.7%) had a comparable nAb titer (median titer 1:50, min 1:17, max: 1:731) as observed in symptomatic non-severe COVID-19 patients within the cohort (Figure 2).

### 3.3. The Live-Virus SNT Assay Comparison Reveals High Sensitivity for Live-Virus SNT Compared to Other Immunoassays

**Figure 3:**
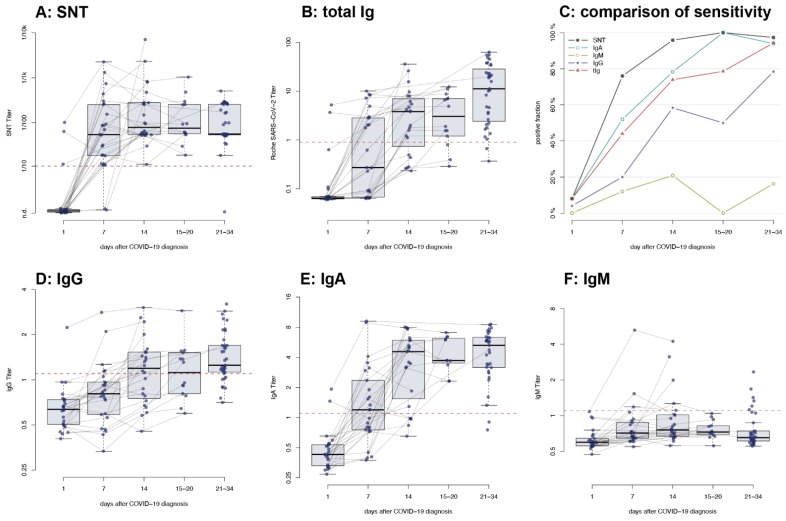
Time course of antibody titers of patients with non-severe COVID-19 measured on d1, d7, d14 and d30 post diagnosis: (**A**) neutralizing antibodies measured by serum neutralization test (SNT); (**B**) total SARS-CoV-2-specific antibodies measured by electrochemiluminescence immunoassay (ECLIA), (**D**) immunoglobulin (Ig) G, (**E**) IgA, and (**F**) IgM; (**C**) sensitivity of each test at the separate time points post-diagnosis. Dashed line: limit of detection as indicated by the manufacturer (**B**,**D**–**F**) or as determined by the experimental setting (**A**). n.d. = not detected.

The serological response to SARS-CoV-2 was assessed in 24 patients. As previously mentioned, all patients developed a measurable nAb titer within 14 days of diagnosis (Figure 3A), and most patients developed an immune response measurable by electrochemiluminescence immunoassay (ECLIA, Figure 3B). Immunoglobulins (Ig) G and IgA could be detected in most patients, with the IgA response preceding IgG (Figure 3D,E). Interestingly, IgM was only detected in a minority of patients even after 14 days (Figure 3F). When compared, the serum neutralization test (SNT) was the most sensitive test for the detection of SARS-CoV-2-specific antibodies at the early stage of infection and maintained a high sensitivity throughout the entire study period (Figure 3C).

### 3.4. Clinical Presentation Does Not Correlate with Neutralizing Antibody Response in Young Adults with Non-Severe COVID-19

**Figure 4:**
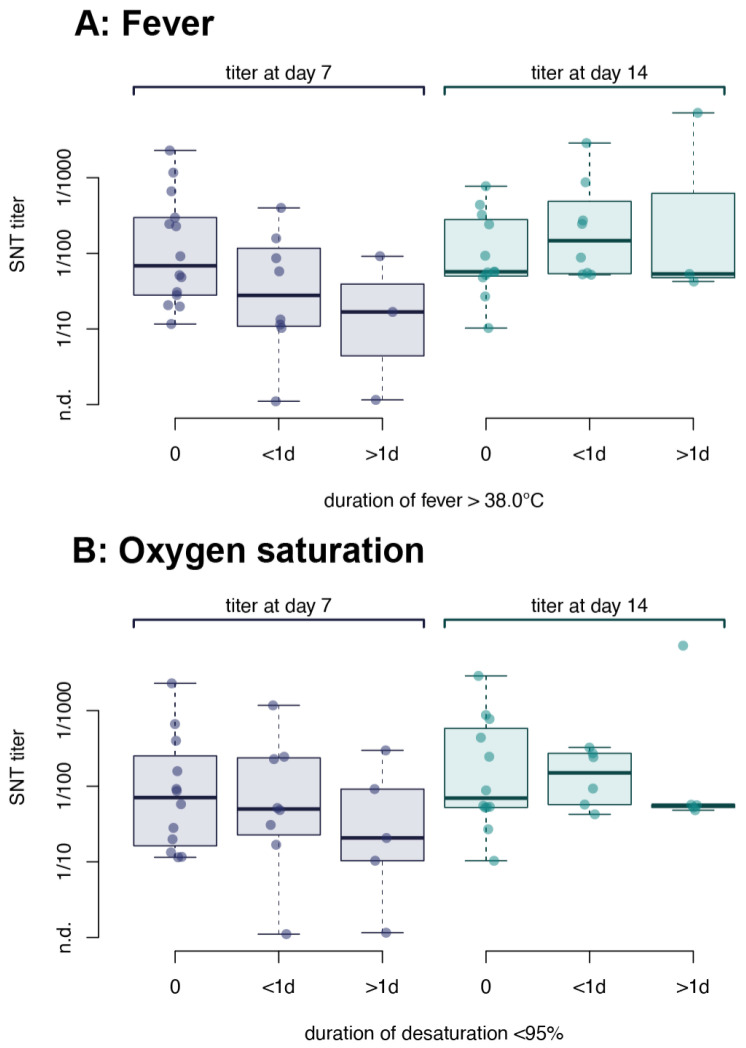
Correlation of the severity of COVID-19 with the antibody response in young adults. Neutralizing antibody titers were correlated with (**A**) tympanic temperature and (**B**) oxygen saturation of symptomatic patients. Patients were grouped according to fever: no fever (never > 38.0 °C), fever < 24 h or fever ≥ 24 h or according to oxygen saturation: no desaturation (never < 95% spO_2_), <24 h desaturation or ≥24 h desaturation. Data were stratified according to the day of sampling relative to the day of diagnosis. No significant differences were observed between the groups by analysis of variance (*p* = 0.68 for fever and *p* = 0.66 for desaturation). n.d. = not detected.

We correlated the clinical features of 52 patients diagnosed with COVID-19 to measured nAb titer. No difference was observed between patients without fever, with fever >38 °C for less than a day, or with sustained fever for more than one day (Figure 4A). Several patients had reduced peripheral oxygen saturation (<95%) as measured by oximetry while breathing ambient air (although at an altitude of 1350 m above sea level), some even for several days (Figure 4B). However, we could also not identify a correlation of desaturation with the antibody titer. None of the patients received oxygen supplementation and all recovered within 1–18 days after diagnosis. 

### 3.5. Common Symptoms of COVID-19 Are Not Related to the Quantitative Serological Response

**Figure 5:**
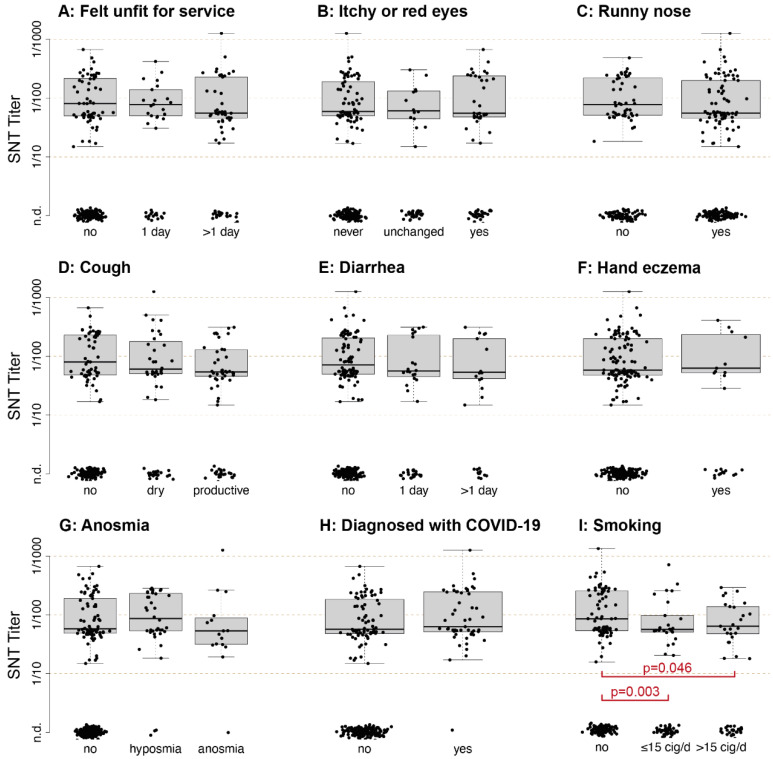
Correlation of symptoms and predisposing factors with the antibody response in young adults. Cross-sectional serum samples from 316 individuals were taken on the same day and neutralizing antibody titers were measured and plotted against self-declared symptoms experienced in the 30 days prior to sampling. Symptoms included general unfit feeling (**A**), itchy or red eyes (**B**), runny nose (**C**), cough (**D**), diarrhea (**E**), hand eczema (**F**), hyposmia or anosmia (**G**). Additionally, no differences in titers could be seen between the symptomatically and asymptomatically infected (**H**). (**I**) shows titers of individuals grouped by their smoking status: Individuals that smoked and produced nAbs showed significantly decreased titers as compared to non-smokers. Statistical significance was determined with the Wilcoxon rank sum test. *p*-values < 0.05 were considered statistically significant. n.d. = not detected.

We compared the nAb titers of participants to symptoms reported in a structured questionnaire administered in the 30 days prior to sampling. These 30 days virtually covered the whole outbreak, with the first patient being diagnosed on 11 March 2020. Although participants that reported having felt unfit for military service had a higher rate of detectable nAbs (unfit for 1 day: 57%, 21/37, >1 day: 68%, 45/66) than the ones not having felt unfit (30%, 63/213), no difference in neutralizing titers could be observed between the three groups if only accounting for participants with detectable nAbs (Figure 5A). Neither self-reported conjunctivitis (Figure 5B), rhinitis (Figure 5C), cough (Figure 5D), diarrhea (Figure 5E), hand eczema (Figure 5F) nor hyp-/anosmia (Figure 5G) had a significant influence on the observed neutralizing titers. When comparing all participants previously diagnosed with COVID-19 with their non-diseased counterparts, again no difference in titers could be detected (Figure 5H). However, having experienced symptoms common for COVID-19 within 30 days prior to sampling significantly increased the likelihood of producing antibodies against SARS-CoV-2 (Table 1).

### 3.6. Smokers Produce Significantly Lower Levels of nAbs Compared to Non-Smokers

Additionally, we aimed to provide additional data on predisposing factors associated with neutralizing antibody production. Neither body mass index (BMI) nor smoking habits had any significant impact on seroconversion rate, and other predisposing factors such as asthma, hay fever, or influenza vaccination in the past season did not influence the ability of volunteers to produce antibodies (Table 2). However, the antibody levels of smokers that did have nAbs were significantly lower than the levels of non-smokers (Figure 5I), independent of the number of cigarettes smoked per day.

## 4. Discussion

In the current study, we evaluated the production of SARS-CoV-2-specific neutralizing antibodies (nAbs) in a large and homogeneous cohort of 332 young, predominantly male adults and detected nAbs in 51 out of 52 patients that had suffered from non-severe COVID-19 and 80 asymptomatically infected individuals. Neutralizing antibody titers did not differ between the two groups (Figure 2), suggesting that asymptomatic infection with SARS-CoV-2 can induce an immune response comparable to non-severe COVID-19. However, in the asymptomatic group, there were individuals with detectable viral RNA in the nasal cavity without nAb. It is unclear if these individuals go on to produce antibodies once they progress through the infection. Additionally, within our cohort of non-severe COVID-19, we did not observe any attenuation of nAb titers throughout the month of available data.

Given the incidence of non-severe and asymptomatic infections in the current SARS-CoV-2 pandemic, insufficient antibody production after such infections coupled with potential antibody waning over time, could result in re-infections at a later time point [12]. However, the severity of the disease would likely be mitigated due to the protective properties of other elements of the immune system [13] and due to the potential presence of memory B-cells being able to swiftly ramp up nAb production in the case of a re-infection. Current studies on the antibody response to SARS-CoV-2 have reported similar patterns as observed for the other two zoonotic CoVs where the majority of infected individuals seroconvert and it has previously been reported that long-lasting (>1 year) functional antibodies against both Middle East Respiratory Syndrome (MERS)-CoV and the first SARS-CoV have been detected in survivors. However, there is consistent evidence of antibody waning over time. In contrast to SARS-CoV-2, the first SARS-CoV was mainly associated with symptomatic disease and although nAb titers are detectable in SARS survivors up to 12 years post-infection, their neutralizing capacity is reduced and likely to be non-protective [14,15,16,17,18]. More importantly, antibody responses after non-severe or asymptomatic MERS-CoV infections are limited or decline rapidly after recovery [19,20,21,22]. Supporting this, SARS-CoV-2 specific nAb titers have been shown to be much higher in patients requiring intensive care as compared to patients with a milder course of the disease [23]. Thus, antibody titers seem to be determined by disease severity and not duration, with varied numbers of asymptomatic cases having been reported to produce nAbs [24]. This, however, does differ from what was observed for the first SARS coronavirus, where a higher neutralizing antibody response correlated with shorter illness duration [25].

To date, individuals with mild or subclinical symptoms account for roughly 80% of those infected with SARS-CoV-2 [26], making it crucial to understand their antibody response. We show that 7 days post infection, most individuals have developed a robust immune response and produce antibodies capable of neutralizing the virus (Figure 3A). This early antibody response predominantly seems to be composed of IgA antibodies (Figure 3E), secreted from mucosal membranes, while IgG are produced later (about 14 days post infection, Figure 3D). Interestingly, an IgM response is observed only sporadically in this cohort (Figure 3F), which differs from previous findings [22] but might be explained by the age of our cohort and the course of disease since higher antibody levels have generally been observed in older and hospitalized patients [27]. Additionally, the immunoassays used for the different antibody classes are not from the same manufacturer, and thus, the studied epitope varies accordingly. While the ELISA used to detect IgA targets the spike antigen, the ELISAs used to detect both IgG and IgM target the nucleocapsid, the same epitope used in the ECLIA. Thus, we cannot exclude a confounding effect on antibody class by different epitopes targeted at different time points with the current data.

Furthermore, we wondered if individual symptoms of COVID-19 could correlate with a higher antibody titer. During cross-sectional testing, we assessed the symptomatology of all participants by a structured questionnaire. Although the symptoms typical for COVID-19 such as fatigue, conjunctivitis, rhinitis, cough, diarrhea are significantly correlated with the detection of neutralizing antibodies (Table 1), titers of those who produced nAbs did not appear to correlate with these symptoms (Figure 5A–G), which corroborates our observation that symptomatology does not correlate with antibody titers in our cohort (Figure 2 and Figure 5H). When studying predisposing factors for infection, neither body mass index (BMI), smoking status, previous vaccination to influenza, or hay fever correlated with the occurrence of nAbs (Table 2), therefore these factors thus most likely do not predispose individuals to infection with SARS-CoV-2. However, when studying titers of those who produced nAbs, we found significantly lower titers in smokers as compared to non-smokers, and this correlation was independent of the amount of cigarettes smoked per day. Smokers have been shown to respond with lower nAb titers to a variety of respiratory pathogens [28] and this finding could explain the previously reported more severe course of COVID-19 in smokers but a non-changed infection rate overall [29].

During clinical observation, we identified different clinical courses in the cohort. Not all patients developed a fever and we found no significant correlation between either body temperature or oxygen saturation and observed nAb titers (Figure 4A,B). Since young adults rarely develop a fever during the course of COVID-19 [30] and only three of our patients suffered from fever for longer than 24 h, our sample size might be too small to detect an effect of fever on nAb titers.

Taken together, our data show that asymptomatic infections with SARS-CoV-2 are able to induce similar immunity as non-severe COVID-19. However, it remains to be clarified if such asymptomatic or mildly symptomatic infections induce a long-lasting immune response and protection while it has been shown that patients suffering from severe COVID-19 exhibit a robust immune response, including a high nAb titer, presumably resulting in protection from re-infection [31]. More data needs to be collected to answer those questions and long-term follow-up studies of different population groups might provide more insight into specific immunological responses and their therapeutic implications over time.

## Figures and Tables

**Table 1:** Symptoms of infection with SARS-CoV-2.

Symptom		Without nAb	With nAb	Statistics
Conjunctivitis	no/unchanged	155	98	*p* = 0.047 OR = 1.8
	yes	95	34	
Cough	no	138	59	*p* < 10^−6^ OR = 3.3
	yes	49	79	
Feeling unfit for service	no	150	63	*p* < 10^−8^ OR = 4.2
	yes	37	66	
Diarrhea	no	155	92	*p* = 0.02 OR = 1.9
	yes	32	37	
>1 of the above symptoms	no	157	88	*p* < 0.001 OR = 2.5
	yes	29	41	

Symptoms within the last 30 days. *p*-values calculated with Fisher’s exact test. OR = odds ratio.

**Table 2:** Predisposing factors and neutralizing antibodies.

Symptom		Without nAb	With nAb	Statistics
BMI	Median (25–75%)	22.8 (20.8–25.2)	23.0 (21.5–25.9)	n.s.
Smoker	non-smoker	113	79	n.s.
	<15 cig/day	46	25	
	≥15 cig/day	28	25	
Influenza vaccination in the past season	no	94	58	n.s.
	yes	62	52	
Asthma	never	159	112	n.s.
	prior/no therapy	17	12	
	yes/treated	10	5	
Hay fever	no	128	94	n.s.
	yes	59	36	

*p*-values calculated using Fisher’s exact test, except for BMI, where significance was calculated using the Welch Two-Sample t-test. n.s. = not significant (*p* > 0.05).

## Data Availability

The data presented in this study are available as far as anonymity of the participants can be guaranteed from the corresponding authors upon reasonable request. The data are not publicly available to prevent identification of individual participants.
